# Genetic Diversity and Geographic Spread of Henipaviruses

**DOI:** 10.3201/eid3103.241134

**Published:** 2025-03

**Authors:** Yakhouba Kane, Betty Nalikka, Alexander Tendu, Victor Omondi, Kathrina Mae Bienes, Abdou Padane, Veasna Duong, Nicolas Berthet, Gary Wong

**Affiliations:** Shanghai Public Health Clinical Center, Fudan University, Shanghai, China (Y. Kane); Institut Pasteur, Phnom Penh, Cambodia (B. Nalikka, A. Tendu, V. Omondi, K.M. Bienes, V. Duong, G. Wong); Institut de Recherche en Santé, de Surveillance Épidémiologique et de Formation, Dakar, Senegal (A. Padane); Institut Pasteur, Paris, France (N. Berthet); Institut Pasteur, Vientiane, Laos (G. Wong)

**Keywords:** Henipaviruses, viruses, genetic diversity, zoonoses, meningitis/encephalitis, paramyxoviridae, bats, shrews, respiratory infections

## Abstract

Henipaviruses, such as Hendra and Nipah viruses, are major zoonotic pathogens that cause encephalitis and respiratory infections in humans and animals. The recent emergence of Langya virus in China highlights the need to understand henipavirus host diversity and geographic spread to prevent future outbreaks. Our analysis of the National Center for Biotechnology Information Virus and VIRION databases revealed ≈1,117 henipavirus sequences and 142 complete genomes. Bats (64.7%) and shrews (11.7%) dominated the host species record, and the genera *Pteropus* and *Crocidura* contained key henipavirus hosts in Asia, Australia, and Africa. Henipaviruses found in the *Eidolon* bat genus exhibited the highest within-host genetic distance. Phylogenetic analysis revealed batborne and rodent- or shrew-derived henipaviruses diverged ≈11,000 years ago and the first known lineage originating in *Eidolon* genus bats ≈9,900 years ago. Pathogenic henipaviruses diverged from their ancestors 2,800–1,200 years ago. Including atypical hosts and regions in future investigations is necessary to control future outbreaks.

Henipaviruses belong to the family *Paramyxoviridae*, a group of enveloped, single-stranded RNA viruses (*1*). During the past 3 decades, henipaviruses have gained considerable attention because of their zoonotic potential, causing severe and often fatal encephalitis and respiratory disease in humans and animals (*2*). Outbreaks caused by Hendra virus (HeV) and Nipah virus (NiV) were linked to bats and are particularly deadly to humans, exhibiting case-fatality rates of 75% for HeV infection and 40%–80% for NiV infection (*3*,*4*). The emergence of HeV in Australia in 1994 and of NiV in Malaysia during 1998–1999, Bangladesh and India beginning in 2001, and the Philippines in 2014 demonstrated the viruses’ ability to infect humans and various domestic animals, causing devastating effects (*5*–*12*).

Henipaviruses ecology and distribution patterns rely on reservoir host circulation, with spillover leading to sporadic outbreaks (*13*,*14*). Studies focusing on pteropid bats revealed diverse henipaviruses and henipa-like viruses in South and Southeast Asia, China, Australia, and Africa and recently in Europe and South America (*5*,*15*–*22*). Most HeV and NiV infections in humans come from contact with contaminated fruits or domestic animals (*13*,*23*). The broad distribution of henipaviruses and discovery of new hosts suggests inconsistent surveillance and unidentified potential hosts.

The discovery of emerging henipaviruses, such as Mojiang virus (MojV) and Langya virus (LayV), highlights the threat to humans might extend beyond HeV and NiV (*20*,*24*). MojV is a nonbat henipavirus that was detected in a cave-rat in the Yunnan Province of China after 3 miners died in 2012 from a severe pneumonia with unknown etiology (*24*). LayV, a recently discovered shrewborne henipavirus, was detected in febrile patients in China in 2018, and spillover events were estimated to have occurred during 2018–2022 (*20*). In Africa, molecular and serologic data supported the circulation of henipaviruses in bats and domestic animals, with evidence of spillover into humans without observable clinical disease (*16*,*25*,*26*).

Efforts to develop henipavirus vaccines and antiviral drugs led to promising candidates in various stages of development, including DNA- and mRNA-based vaccines and neutralizing antibody products (*27*–*30*). However, no licensed vaccine is available for human use, and treatments remain limited to supportive care. Since 2012, only the Equivac vaccine is licensed for horses in Australia (*31*). The growing threat of henipaviruses and the possibility of human-to-human transmission underscore the importance of studying henipavirus host distribution and assessing outbreak risks (*7*).

In this study, we aimed to increase understanding of the henipavirus host spectrum and distribution patterns by analyzing existing data from public repositories. We focused on henipavirus infections in nonhuman mammals to assess the spatial distribution of these viruses and the diversity of their associated hosts. We further assessed the origin, diversification, and cross-species transmission of henipaviruses. In this article, we have defined the term reservoir or reservoir host as the animal species that repeatedly tested positive for henipavirus, shed infectious viruses, and supported long-term viral maintenance across locations. We have defined an accidental host as an animal species that tested positive for henipavirus but does not necessarily support its sustained transmission or maintenance, often acting as a dead-end host.

## Materials and Methods

### Data Collection and Processing

We searched for available sequence data of the family *Paramyxoviridae* in the National Center for Biotechnology Information (NCBI) Virus database (https://www.ncbi.nlm.nih.gov/labs/virus) on December 11, 2023, by using the keyword “Paramyxoviridae,taxid:11158” and downloaded the results. We downloaded the sequence metadata and included the columns relevant for this study (Appendix Table 1). We removed the rows corresponding to accessions without confirmed host and laboratory generated sequences. We excluded animal-derived henipavirus sequences <100 bp from the analysis because of frequent lack of host or country information. We collected host data for henipaviruses from the VIRION database, the atlas of vertebrate viromes (*32*). We removed records from the analysis that were not taxonomically resolved to the NCBI backbone or had uncertainty in host identification.

We performed a descriptive analysis of henipavirus hosts by using R packages dplyr and ggplot2 v.3.5.1 (https://ggplot2.tidyverse.org). This analysis included filtering and summarizing the metadata, saving unique host data, and creating visualizations, including a temporal trend of sequence submission and a heatmap to represent host species distribution. We created a choropleth map visualizing total henipavirus sequences by country and the number of unique host genera of henipavirus across countries by using Python packages geopandas (Zenodo, https://zenodo.org/records/3946761), matplotlib, and pandas (Python, https://pandas.pydata.org) to filter and group metadata and merge with world shapefile.

### Evolutionary Divergence and Spread of Henipaviruses

We screened a total of 167 henipavirus sequences (>14,000 bp), including 142 complete genomes, to filter out sequences with unknown nucleotides >0.05% and aligned by using MAFFT version 7.505 with the default parameters (*33*). We trimmed the alignment by using TrimAl 1.2rev57 and the duplicated sequences dropped with Seqkit version 2.8 (*34*,*35*). We used ModelFinder implemented in IQ-TREE2 to detect the best-fit model (*36*). To assess the relatedness of henipaviruses across host groups, we computed the mean genetic distance of henipaviruses within and between host genera by using MEGA version 11.0.13 (https://www.megasoftware.net) with the following settings: 500 bootstrap replications, Kimura 2-parameter model, gamma distributed with gamma parameter set to 4 (*37*).

We performed a Bayesian time-resolved phylogeny and ancestral host reconstruction by using BEAST version 1.10.4 with host genus and country as discrete characters (*38*). We used Bayesian discrete phylogeographic method implemented in BEAST to construct the ancestral hosts. Because we found no temporal signature, BEAST analysis was based on a fixed substitution rate of 1.0 substitutions per site per unit of time. We ran the analysis for a total of 200 million iterations and collected samples every 20,000 generations. We used the Hasegawa-Kishino-Yano substitution model, a strict molecular clock, and a constant size coalescent prior (*39*). We chose those settings to ensure comprehensive sampling of the posterior distribution. We assessed convergence of the Markov Chain Monte Carlo chains by using Tracer version 1.7, where parameters were checked for sufficient effective sample sizes and the first 10% of iterations were discarded as burn-in (*40*). We used the remaining samples to generate maximum clade credibility trees, which were visualized by using FigTree version 1.4.4 (http://tree.bio.ed.ac.uk/software/figtree) to interpret the phylogenetic relationships and divergence times within the dataset. In addition, we performed ancestral sequence reconstruction by using the empirical Bayesian method implemented in IQ-TREE 2.3.2 with the following parameters: the ModelFinder Plus model selection, DNA sequence type, ancestral sequence reconstruction, and 1,000 ultrafast bootstrap replicates (*41*). This method enables the reconstruction of the most likely ancestral states at each node of the tree, accounting for model uncertainty and providing robust support for inferred ancestral sequences.

## Results

### Temporal Trend and Major Host Groups of Paramyxoviruses

**Figure 1:**
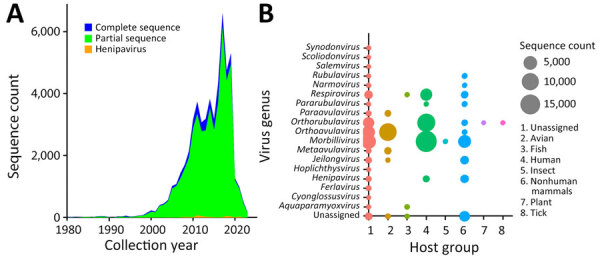
Trend in paramyxovirus sequences submitted to the National Center for Biotechnology Information Virus database (https://www.ncbi.nlm.nih.gov/labs/virus), 1980–2023. A) Sequence count by collection year, showing all complete and partial sequences compared with all henipaviruses. B) Virus genera and sequence counts by major host group from the VIRION database (*32*).

We visualized the temporal trend of paramyxovirus sequences in GenBank (Figure 1, panel A). A total of ≈69,000 paramyxovirus sequences were identified, and 8.6% (n = 5,841) were complete genomes. Sequences of *Morbillivirus*, *Orthorubulavirus*, and *Respirovirus* are predominantly human-associated, whereas *Orthoavulavirus* sequences primarily originate from avian hosts and henipavirus sequences primarily originate from nonhuman mammal hosts (Figure 1, panel B). Since 2002, paramyxovirus sequences have increased exponentially, with noted reductions in 2012, 2015, 2018, and 2020 (Figure 1, panel A). Henipaviruses accounted for ≈1,117 nucleotide sequences, including 859 from nonhuman mammals. Among the 142 complete henipavirus genomes recorded, 90 originated from nonhuman animals, largely from the genera *Crocidura* and *Pteropus* (*20*).

### Henipavirus Host Range

**Figure 2:**
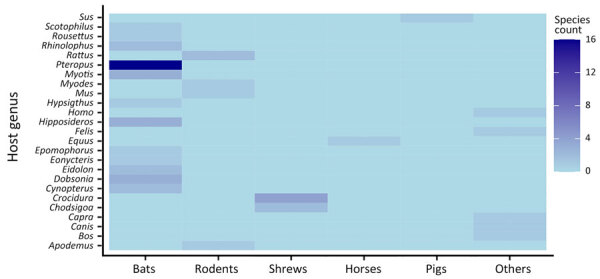
Numbers of henipavirus species by host group for sequences submitted to the National Center for Biotechnology Information Virus database (https://www.ncbi.nlm.nih.gov/labs/virus), 1980–2023. Host groups from the VIRION database (*32*) are represented at the genus level.

**Figure 3:**
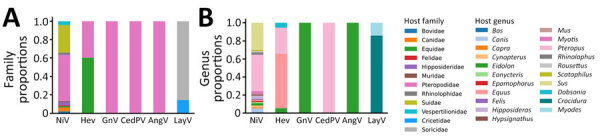
Proportional counts of henipaviruses by host family and genus for sequences submitted to the National Center for Biotechnology Information Virus database (https://www.ncbi.nlm.nih.gov/labs/virus), 1980–2023. A) Virus detection proportions across host families. B) Virus detection proportions across host genera. AngV, Angavokely virus; CedPV, Cedar virus; GnV, Ghana virus; HeV, Hendra virus; LayV, Langya virus; NiV, Nipah virus.

Henipaviruses showed the potential to infect diverse mammalian taxonomic groups. Our analysis revealed 668 henipavirus records involving 51 unique mammal species distributed among 25 genera and 13 families (Figures 2, 3; Appendix Table 2). Most henipavirus host records were associated with wild mammals and bats and shrews identified as the key animal group hosts. Although henipavirus detection spans 51 species, not all species act as competent hosts with virus-shedding and transmission capabilities. 

The proportions of henipavirus detection rate across host families and genera vary (Figure 3). Bats (order Chiroptera) represent the most diverse host group for henipaviruses, comprising 64.7% of the total host species (Figure 2; Appendix Table 2). Those records involve 4 bat families: Hipposideridae, Pteropodidae, Rhinolophidae, and Vespertilionidae. Of note, ≈65% of NiV detection involved the Pteropodidae family, and other bat families had a relatively low detection rate: 5.6% for Hipposideridae, 3.7% for Rhinolophidae, and 6.6% for Vespertilionidae (Figure 3, panel A).

The family Pteropodidae is the most diverse, encompassing 8 genera previously known to host henipaviruses: *Cynopterus*, *Dobsonia*, *Eidolon*, *Eonycteris*, *Epomophorus*, *Hypsignathus*, *Pteropus*, and *Rousettus*. Within those genera, henipaviruses were detected in 25 species, with the genus *Pteropus* displaying detection records of 28.8% for HeV and 40.3% for NiV, involving various species such as *P. vampyrus*, *P. hypomelanus*, and *P. medius* (formerly *P. giganteus*) (Figure 3, panel B). The family Vespertilionidae comprised 2 genera, *Myotis*, with a detection record of ≈3%, and *Scotophilus*, with a detection record of <2%, and 3 species, *Myotis daubentonii*, *Myotis ricketti*, and *Scotophilus kuhlii*. The family Hipposideridae includes the single genus *Hipposideros*, accounting for 3.7% of NiV instances, with 3 involved species: *H. armiger*, *H. larvatus*, and *H. pomona*. The family Rhinolophidae consists of the genus *Rhinolophus* and exhibits 2.5% NiV detection records with 2 species: *R. affinis* and *R. sinicus*.

Shrews (family Soricidae) have emerged as a major group of *Henipavirus* hosts, accounting for 11.7% of recorded henipavirus host species (Figures 2, 3; Appendix Table 2). They are distributed across 2 genera: *Chodsigoa*, with 2 species (*C. hypsibia* and *C. smithii)*, and *Crocidura*, with 4 species (*C. attenuata*, *C. lasiura*, *C. shantungensis*, and *C. tanakae*). Of note, >85% of LayV instances were recorded in shrews of the genus *Crocidura*.

Rodents (order Rodentia) represented ≈9.8% of henipavirus host species records, involving 2 families, Cricetidae and Muridae. The family Muridae is more diverse, comprising 3 genera: *Apodemus*, *Mus*, and *Rattus*. The identified species within those genera are *A. agrarius*, *M. musculus*, *R. rattus*, and *R. tanezumi*. The family Cricetidae includes the genus *Myodes*, and the single species *M. rutilus* is associated with >14% of LayV detection record.

Our data revealed a spectrum of domestic animals with evidence of henipavirus infection. Bovids (Bovidae) and swine (Suidae) each accounted for ≈3.9% of the host species records. Bovines encompassed 2 genera: *Bos* (*B. taurus*) and *Capra* (*C. hircus*), with 3.7% of NiV instances, whereas swine are represented by the single genus, *Sus* (*S. scrofa* and *S. crofa domesticus*), accounting for 6.6% of NiV.

Canids (Canidae), equids (Equidae), and felids (Felidae) each represent ≈1.9% of the host species records. Canids are represented by the genus *Canis* and the species *C. lupus*, in which 2.8% NiV instances were identified. Equids included the single genus *Equus*, specifically *E. caballus* (the domestic horse). Of note, HeV was predominantly detected in domestic horses, with over 72.8% occurrences, compared with 1.8% for NiV. Felids were represented by the genus *Felis*; the species *F. catus* (the domestic cat) was involved in 1.8% of NiV instances.

### Geographic Distribution of Henipavirus Hosts

**Figure 4:**
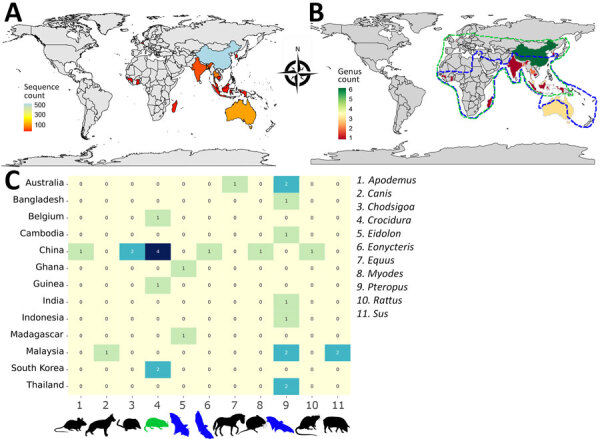
Geographic distribution of henipavirus hosts by country on the basis of henipavirus sequences in the National Center for Biotechnology Information Virus database (https://www.ncbi.nlm.nih.gov/labs/virus) as of December 11, 2023. A) Number of henipavirus sequences in each country. B) Henipavirus host diversity at country level, estimated by calculating the sum of animal genera per country. The habitat range of pteropodids is circled in blue and the shrew genus *Crocidura* in light green. C) Henipavirus host species for each animal genus across countries.

Our analysis is on the basis of henipavirus sequence records across many countries from the NCBI Virus database. The dataset comprises information on the geographic occurrence of potential henipavirus hosts. We identified a total of 806 of 859 henipavirus records involving diverse animal host species spanning ≈11 mammal genera from 13 countries (Figure 4). Although the presence of henipavirus sequences in a host species may indicate exposure or infection, the reservoir status for many of those hosts remains unverified.

China contributed >50% (446) of henipavirus sequence records with a diverse array of hosts including 1 bat genus (*Eonycteris*), 3 rodent genera (*Apodemus*, *Myodes*, *Rattus*), and 2 shrew genera (*Chodsigoa*, *Crocidura*). Of note, most of those records involve shrews, particularly the genus *Crocidura* and the species *C. lasiura*, suggesting those small mammals are major henipavirus carriers in China. In addition, 2 shrew species, *C. lasiura* and *C. shantungensis*, were found infected with henipaviruses in South Korea.

In mainland Southeast Asia, bat species of the genus *Pteropus* emerged as a primary host group for henipaviruses. In Bangladesh and India, the Indian flying fox (*P. medius*) is the reservoir of henipaviruses. Henipavirus instances in Malaysia were linked to 2 *Pteropus* species, *P. hypomelanus* and *P. vampyrus*, although some occurrences were noted in domestic animals including pigs (*S. scrofa*, *S. scrofa domesticus*) and dogs (*C. lupus familiaris*). In Indonesia, the large flying fox (*P. vampyrus*) was identified as a henipavirus host. In Cambodia and Thailand, henipavirus occurrences were primarily associated with the common fruit bat (*P. lylei*). *P. hypomelanus* bats also contributed to a small portion of henipavirus records in Thailand.

In Australia, henipaviruses are primarily linked to bat species within the genus *Pteropus*. The black flying fox (*P. alecto*), the spectacled flying fox (*P. scapulatus*), and the gray-headed flying fox (*P. poliocephalus*) are prominent reservoir host species for henipaviruses in this region. In addition, equids such as *E. caballus* are henipavirus hosts of note in this region.

In Africa, we identified the bat genus *Eidolon* as a critical host group of henipaviruses. In Madagascar, the Madagascan fruit bat (*E. dupreanum*), an endemic and vulnerable species, is a prominent host for henipaviruses. In West Africa, henipaviruses occurrences were noted in the straw-colored fruit bat (*E. helvum*) in Ghana and in the shrew species *C. grandiceps* in Guinea. In Europe, henipaviruses occurrence has also been confirmed with another shrew species, *C. russula*, as the only identified host in Belgium.

### Evolutionary Divergence and Cross-Species Transmission of Henipaviruses

**Figure 5:**
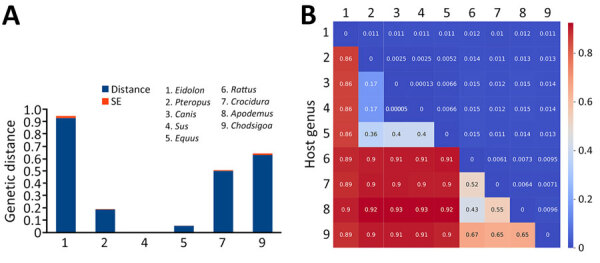
Evolutionary divergence and spread of henipaviruses for sequences submitted to the National Center for Biotechnology Information Virus database (https://www.ncbi.nlm.nih.gov/labs/virus), 1980–2023. A) Genetic distance of henipaviruses within host genera. B) Genetic distance of henipaviruses between host genera. SE, standard error.

We investigated the evolutionary distances of henipaviruses within and between their diverse hosts (Figure 5, panels A, B). Henipaviruses from the bat genus *Eidolon* exhibited the highest within-host genetic distance (D = 0.92), followed by those from the shrew genera *Chodsigoa* (D = 0.62) and *Crocidura* (D = 0.49) (Figure 5, panel A). In contrast, the bat genus *Pteropus* showed relatively low within-host genetic distance (D = 0.18) for henipaviruses, and henipaviruses from swine and equid showed almost no diversity (D<0.1). The analysis of the genetic distances of henipaviruses between host groups showed a clear dichotomy between small nonflying and flying mammal henipaviruses (Figure 5, panel B). Rodents and shrews shared more closely related henipaviruses, whereas bats of the genus *Pteropus* had henipaviruses similar to those found in domestic animals. Of note, henipaviruses from *Eidolon* genus bats appeared distantly related to all other host groups.

**Figure 6:**
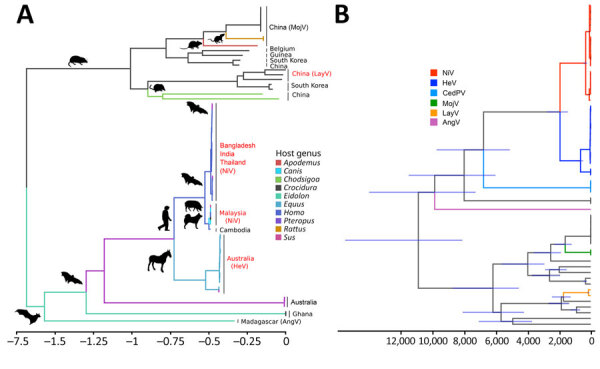
Time-calibrated phylogenetic trees showing the evolutionary divergence and spread of henipaviruses for sequences submitted to the National Center for Biotechnology Information Virus database (https://www.ncbi.nlm.nih.gov/labs/virus), 1980–2023. A) Ancestral host tree demonstrating divergence of hosts and countries of origin. Scale bar indicates relative number of substitution events per site per unit of time. B) Time-calibrated Bayesian phylogeny showing the divergence times for henipaviruses. The node bars indicate 95% HPD. The divergence between batborne and shrewborne henipaviruses occurred ≈11,000 (95% HPD 15,500–8,200) years ago. AngV, Angavokely virus; CedPV, Cedar virus; HeV, Hendra virus; HPD, highest posterior density; LayV, Langya virus; MojV, Mojiang virus, NiV, Nipah virus.

We performed ancestral host reconstruction of henipaviruses by using both a Bayesian discrete phylogeographic approach in BEAST and an empirical Bayesian method in IQ-TREE. Because both methods yielded similar results, we generated the phylogenetic tree from IQ-TREE and a BEAST-derived tree (Figure 6, panels A, B). Phylogenetic analysis supported the results of the genetic distances of henipaviruses, displaying 2 main branches: 1 consisting of batborne henipaviruses and another of rodent- and shrew-derived henipaviruses.

The time-calibrated Bayesian phylogeny supported the divergence of 2 main clades ≈11,000 years ago (95% highest posterior density [HPD] 15,500–8,200 years) (Figure 6, panel B). Considering host genera as discrete character states, henipaviruses likely originated in African fruit bats of the genus *Eidolon* (Figure 6, panels A, B; Appendix Table 1). The Madagascan fruit bat hosts the earliest known henipavirus lineage, other lineages in bats likely emerged from these early lineages ≈9,900 years ago (95% HPD 14,010–7,400 years). Pathogenic henipaviruses, including HeV, LayV, NiV, and MojV, showed a recent divergence from their sister clades ≈2,800–1,200 years ago (Figure 6, panel B). Rodent and shrew henipaviruses displayed an evolutionary origin in the shrew genus *Crocidura*.

Bat-derived henipaviruses, including NiV and HeV, emerged from the bat genus *Pteropus*. The zoonotic transmission of those viruses involved various domestic animals such as horses for HeV and dogs and pigs for NiV, which indicates potential cross-species transmission of henipaviruses (Figure 6, panels A, B). Furthermore, the increased diversity of *Crocidura* shrew henipavirus lineages, along with their close phylogenetic relatedness to other shrew and rodent henipaviruses, suggests shrews might play a critical role as reservoirs and vectors (Figure 6, panels A, B).

## Discussion

In this study, we analyzed the host and geographic range of henipaviruses by using data from public repositories. Henipaviruses showed a broad host range infecting ≈13 mammal families, including bats, rodents, and shrews, predominantly in Africa, Australia, East Asia, South Asia, and Southeast Asia.

Megabats within the genus *Pteropus* displayed a high diversity of henipavirus host species. Because most sampling events targeted pteropodid bats, comprehensive studies are needed to accurately assess the roles of species from other bat families (*42*). Shrews and rodents have emerged as major nonbat hosts, which is critical because of their widespread distribution and ability to host zoonotic pathogens such as hantaviruses and bornaviruses (*43*). Shrews and rodents’ ability to host henipaviruses suggests a broader ecologic and epidemiologic role for those animals than previously recognized, and further study would be beneficial to understanding factors leading to henipavirus maintenance and transmission among these animals.

**Figure 7:**
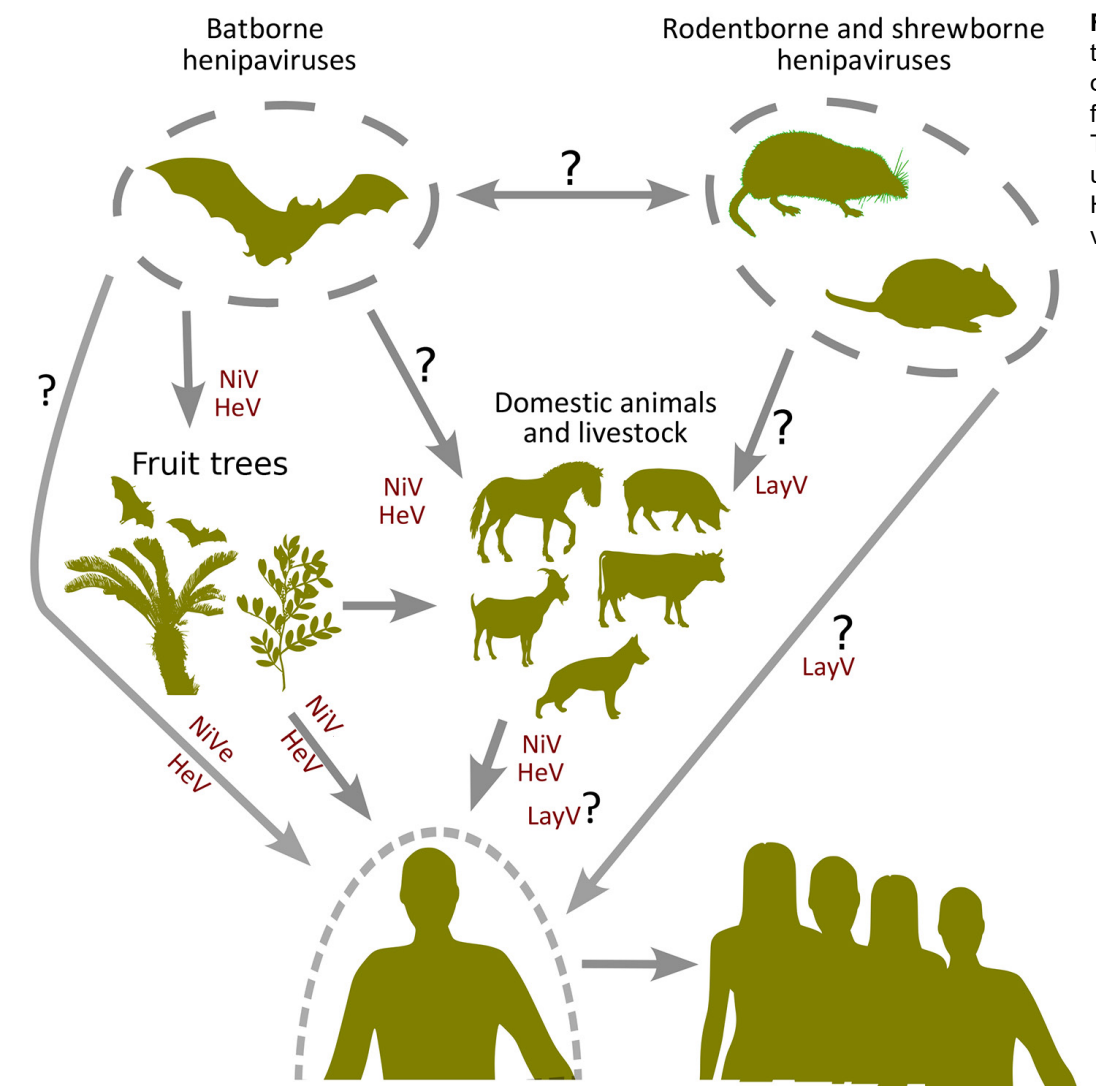
Flowchart showing the potential for host switching of henipaviruses and the routes for potential spillover events. The question marks indicate unconfirmed transmission routes. HeV, Hendra virus; LayV, Langya virus; NiV, Nipah virus.

The geographic spread and discovery of novel nonbat hosts, particularly in China, suggests increased global attention of henipaviruses (*18*,*44*). China likely contains more henipavirus sequence records with a high diversity of nonbat hosts involving shrews and rodents. The emergence of LayV as the first nonbat henipavirus to cause disease in humans indicates potential roles for shrews in zoonotic transmission (Figure 7). Moreover, the prominence and the extensive distribution of shrews, particularly of the genus *Crocidura*, suggests those small mammals as a potential reservoir for henipaviruses in East Asia. Of note, the antigenic profile of LayV and MojV were found to be distinct from NiV, emphasizing the effect of henipavirus diversification along their hosts and the potential difficulty to develop a vaccine that can cover both bat and rodent or shrew derived-henipaviruses (*45*). In addition, the route for the zoonotic transmission of LayV and its geographic extent necessitates further study.

In regions highly affected by pathogenic henipavirus diseases, including South and Southeast Asia and Australia, henipavirus records mostly involve bats of the genus *Pteropus*, although other bat genera may play roles in the maintenance of henipaviruses. Expanding henipavirus sampling to Africa, Europe, and South America has improved understanding of host range, thereby expanding the geographic extent of henipavirus endemicity from its traditionally known regions (*17*–*19*,*25*,*42*–*44*). Even with those efforts, henipavirus studies outside of Asia and Australia remain scarce, potentially overlooking other henipavirus reservoirs.

Henipaviruses from *Eidolon* genus bats showed increased genetic diversity, likely because of their wide geographic distribution and diverse ecologic niches. Rodents and shrews share closely related henipaviruses, whereas bats, particularly from the genus *Pteropus*, harbor henipaviruses related to those found in domestic animals. This relation suggests host-specific adaptations and evolutionary pressures. Ancestral host reconstruction pointed to the African fruit bats (genus *Eidolon*) as the henipavirus origin. The earliest known henipavirus lineage dates back ≈9,900 years, suggesting a longstanding association with African fruit bats (*16*,*45*). The recent divergence of pathogenic henipaviruses aligns with their emergence as major zoonotic threats, emphasizing the dynamic nature of henipavirus evolution and the potential for sudden outbreaks. Rodent and shrew henipaviruses likely originated in *Crocidura* shrews. The close phylogenetic relationship between henipaviruses from those animal groups highlights active host-switching events (Figure 6, panel A). Moreover, the hopping of bat-derived henipaviruses from pteropodid bats to diverse domestic animals underscores the need for monitoring regions with major reservoirs (Figure 7).

Despite increased paramyxovirus data, several abrupt declines during major events (severe acute respiratory syndrome in 2002, Middle East respiratory syndrome in 2012, Ebola and Zika in 2014–2016, Ebola in 2018, and COVID-19 in 2020) were noted (Figure 1, panel A). Those observations suggest the effect of global outbreaks on existing surveillance efforts. The limited number of henipavirus sequences, specifically complete genome sequences, limits the understanding of their diversity and evolution. Of note, despite evidence of zoonotic spillover of henipaviruses in Africa, only 2 full genomes were publicly available during this study.

Public repositories in virus research led to various challenges because of incomplete data and reporting inconsistencies. During data collection, the first marsupialborne henipavirus sequence was not available online (*18*). Regions may be underrepresented because of serologic method or PCR use without GenBank records, as observed in the Republic of the Congo, the Democratic Republic of the Congo, Cameroon, and South Africa (*16*,*44*). Those challenges with public data collection highlight the importance of improving online repositories to provide more comprehensive and accurate information. However, it is crucial to recognize that most of those limitations are consequences of the limited resources and logistic challenges faced by field researchers and affecting their data collection. Because of those challenges, it is difficult to expect standardized collection of surveillance data from all regions. However, focusing efforts on long-term monitoring and including less-explored hosts like shrews and rodents is essential for a better understanding of henipavirus epidemiology. Enhanced collaboration and resource sharing between local and international institutions could help mitigate those challenges and improve the overall quality of henipavirus research.

In conclusion, the concentration of henipavirus data from countries such as China and Australia highlights their laboratory infrastructure and robust surveillance capacities, which enable extensive data collection and sequencing. This data concentration emphasizes the need to increase the capacity of research facilities and surveillance in other regions to achieve a more globally representative understanding of henipavirus dynamics. We stress the importance of noninvasive methods in virological surveillance. Practices such as culling bat populations and destroying their habitats are harmful and unethical. Approaches that protect both conservation efforts and biodiversity are necessary.

Appendix Additional information about genetic diversity and geographic spread of henipaviruses.
